# An Indoor Navigation Algorithm Using Multi-Dimensional Euclidean Distance and an Adaptive Particle Filter

**DOI:** 10.3390/s21248228

**Published:** 2021-12-09

**Authors:** Yunbing Hu, Ao Peng, Biyu Tang, Hongying Xu

**Affiliations:** 1 School of Informatics, Xiamen University, Xiamen 361001, China; yunbinghu@stu.xmu.edu.cn (Y.H.); tby@xmu.edu.cn (B.T.); 2 Artificial Intelligence and Big Data College, Chongqing College of Electronic Engineering, Chongqing 401331, China; hongying8015@126.com

**Keywords:** inertial navigation system, WiFi fingerprint matching, adaptive particle filter, multidimensional Euclidean distance

## Abstract

The inertial navigation system has high short-term positioning accuracy but features cumulative error. Although no cumulative error occurs in WiFi fingerprint localization, mismatching is common. A popular technique thus involves integrating an inertial navigation system with WiFi fingerprint matching. The particle filter uses dead reckoning as the state transfer equation and the difference between inertial navigation and WiFi fingerprint matching as the observation equation. Floor map information is introduced to detect whether particles cross the wall; if so, the weight is set to zero. For particles that do not cross the wall, considering the distance between current and historical particles, an adaptive particle filter is proposed. The adaptive factor increases the weight of highly trusted particles and reduces the weight of less trusted particles. This paper also proposes a multidimensional Euclidean distance algorithm to reduce WiFi fingerprint mismatching. Experimental results indicate that the proposed algorithm achieves high positioning accuracy.

## 1. Introduction

In open outdoor areas, the global satellite navigation system offers reliable positioning accuracy. For example, Baidu Maps and Google Maps provide navigation and positioning for pedestrians and cars. Yet the transmission of obstacle occlusion signals indoors, in tunnels and in other areas greatly attenuates the wireless signal; therefore, a scheme using satellite navigation cannot deliver reliable positioning accuracy. Scholars have thus begun to study indoor navigation solutions [1,2]. Considering the advantages of the high popularity and ease of carrying of smartphones, researchers have done a lot of research on indoor navigation with the help of smartphone platforms. For indoor pedestrian navigation, the positioning accuracy of the meter level has been within the visible range of users, which meets the positioning needs of most ordinary users. Considering smartphones’ popularity and ease of transportation, researchers have carefully considered indoor navigation based on smartphone platforms.

The inertial navigation system (INS) is an autonomous navigation system that does not depend on external information and does not radiate energy to the outside. This system can provide continuous and real-time data on the carrier’s position, attitude, and speed. Its primary disadvantage is the cumulative effect of position error: the longer the running time, the greater the error. Reference [3] shows that the position error of the two-dimensional plane is proportional to the cubic power of time. Various in-depth investigations have been conducted to address cumulative error. When pedestrians walk in regular corridors, Heuristic drift reduction (HDR) and Heuristic drift elimination (HDE) algorithms [4,5] effectively eliminate cumulative error. Reference [6,7] introduced map information and used a map-aided algorithm to eliminate the direction drift of inertial navigation. Alternatively, one can introduce a magnetic field and use the field’s north-pointing characteristic to correct the INS’s direction.

WiFi wireless signals are widely used for positioning. The signal attenuation model and signal fingerprint matching model are two typical positioning techniques. The attenuation model needs to know the WiFi access point’s location in advance and then calculates the user’s location via the least squares method according to the received signal strength [8]. WiFi signals fluctuate with time, and a complex indoor environment (e.g., with walking pedestrians or changing obstacles) can quickly lead to deviation in the propagation model’s parameter estimation along with poor positioning accuracy. The WiFi fingerprint matching model contains two stages: an offline training stage and online positioning stage. In the first phase, the user stores the received signal and corresponding position in the database; in the second phase, collected fingerprints are matched with fingerprints from the database. The position corresponding to the most similar fingerprint in the fingerprint database is often taken as the pedestrian’s position. Considering that WiFi fingerprints are vulnerable to environmental interference, fingerprint mismatching is unavoidable. Magnetic fingerprint matching was introduced in Reference [9], to further reduce position error due to WiFi fingerprint mismatching. Given the two signal sources’ complementary characteristics, an adaptive particle filter is proposed in this paper to integrate INS and WiFi.

## 2. The Innovation and the New Contributions

Most navigation schemes require infrastructure set up in advance in order to improve positioning accuracy. Unlike these schemes, the one proposed in this paper makes use of existing WiFi and inertial sensors. In an indoor environment, WiFi mismatching due to the “jump” fingerprint is the main problem, so the multi-dimensional fingerprint is introduced. Unlike the multi-dimensional dynamic time warping algorithm in Reference [10], the multi-dimensional Euclidean distance algorithm is not only simple to calculate but also has the potential to reduce fingerprint mismatching. The particle weight in the process of particle sampling is thought to be related not only to the observation but also to the historical position of pedestrians.

The main innovations of our work are as follows:

- Because the improved algorithm does not necessitate additional infrastructure, it is low-cost and simple to update software;
- A multi-dimensional Euclidean distance algorithm is proposed to calculate the distance between fingerprints. A multi-dimensional Euclidean distance algorithm using historical fingerprints can reduce the impact of the “jump point" fingerprints, thus reducing fingerprint mismatching;
- To solve the problem of particle degradation, the historical position information is introduced, and the adaptive factor is used to improve the weight of high trust particles and reduce the weight of low trust particles;
- Two smartphones are used to conduct navigation experiments in two navigation areas. The experimental results show that the average positioning accuracy is 1.84 m.

## 3. Related Work

At present, popular localization algorithms include Dead Reckoning (DR) [11], and the geometric algorithm [12], fingerprint algorithm [13], and image algorithm [14]. Different indoor positioning technologies possess unique characteristics. DR based on inertial sensors can provide continuous navigation and positioning but suffers from cumulative error. Geomagnetism exists in nature; thus, no infrastructure needs to be deployed in advance. Geomagnetism can provide navigation direction but is readily disturbed by metal equipment and can easily lead users in the wrong direction. Meanwhile, fingerprint mismatching is a key concern in fingerprint matching. The image algorithm is similarly problematic: it requires intensive computation and is plagued by visual blind spots.

Although a single positioning signal source carries inherent benefits and drawbacks, its positioning performance can be improved by integrating the complementary characteristics of multiple signal sources. For example, several scholars have studied the hybrid positioning fusion algorithm [15,16]. Reference [17] presented a short-range radio frequency and map to eliminate DR’s cumulative error. Reference [18] used an unscented Kalman filter to fuse DR and Ultra Wide Band (UWB). Kang [19] proposed SmartPDR, which adaptively selects a trusted direction source for a magnetometer and a gyroscope. Li [20] applied an adaptive Kalman filter to fuse an INS and magnetic field matching system; the fusion algorithm effectively reduced cumulative error. Furthermore, Reference [21] used an adaptive unscented Kalman filter to integrate the inertial measurement unit, magnetometer, and floor plan to reduce cumulative error. Yu [22] introduced map information and devised a map-based indoor pedestrian navigation algorithm that incorporated map assistance and map-matching algorithms to eliminate cumulative error.

Another common method involves WiFi signals. Using a DR error model as the state equation with WiFi positioning results and DR as observation equations, Li [23] presented an adaptive and robust Kalman filter to integrate DR and WiFi. Zhuang [24] proposed a tightly coupled extended Kalman filter to fuse DR and WiFi signals; this algorithm could effectively eliminate cumulative error in pedestrian navigation. By adding equality constraints and expanding the distribution of observation noise, a robust constrained Kalman filter has also been proposed to improve DR and WiFi indoor positioning [25].

Inspired by the above work, this paper presents an improved particle filter algorithm combining DR and WiFi. The DR model is taken as the state equation, and the difference between WiFi and DR represents the observation equation for the particle filter. An adaptive particle filter is also proposed to mitigate particle degradation in the traditional particle filter.

## 4. Framework Description

**Figure 1:**
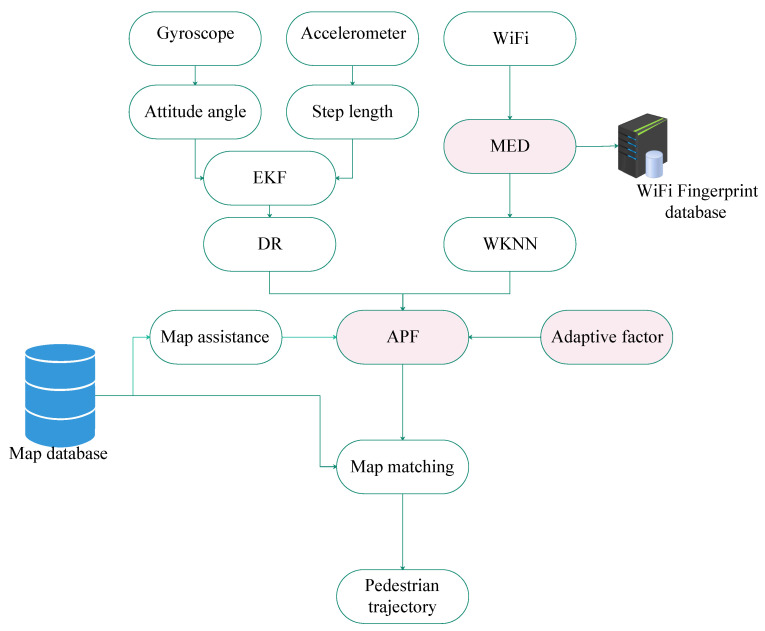
Algorithm framework of MED and APF.

The gyroscope and accelerometer of the smartphone collect angular velocity and acceleration. Using the integral technique obtains the attitude angle. Using the gait technique obtains the pedestrian step length. DR based on direction and step length is used to estimate the position of pedestrians. The process of WiFi fingerprint localization can be divided into the offline fingerprint database construction stage and the online fingerprint matching stage. The reference points of the positioning area are calibrated in advance, and the fingerprints corresponding to the reference points are collected at the same time. The interpolation technology is used to construct the fingerprint database in the positioning area. In the online phase, MED is used to calculate the distance between the WiFi fingerprints collected online and the fingerprints in the database. The weighted K nearest neighbor (WKNN) algorithm further improves the positioning performance by selecting K WiFi matching results. The DR model is used as the state transfer equation of the particle filter, and the difference between the positioning results of WiFi and inertial navigation is used as the observation equation. An adaptive factor is proposed to improve the weight of highly trusted particles. At the same time, map information is introduced as an auxiliary means to eliminate particles crossing the wall. If the particle crosses the wall, its corresponding weight is set to zero. Finally, map matching is used to further reduce the pedestrian position error. The algorithm framework of MED and APF is shown in Figure 1.

### 4.1. DR Module

#### 4.1.1. Attitude Angle

When the gyroscope obtains the angular velocity, the increment of the three-axis angular velocity $\left[\Delta \theta_{k}^{x},\Delta \theta_{k}^{y},\Delta \theta_{k}^{z}\right]$ velocity can be calculated as follows [26]:

(1) $$\begin{array}{cc} \Delta \theta_{k}^{x} & =\omega_{k}^{x}*T_{s}\\ \Delta \theta_{k}^{y} & =\omega_{k}^{y}*T_{s}\\ \Delta \theta_{k}^{z} & =\omega_{k}^{z}*T_{s} \end{array}$$

where $\left[\omega_{k}^{x},\omega_{k}^{y},\omega_{k}^{z}\right]$ represents the angular velocity of the three axes, respectively; and $T_{s}$ represents the sampling interval.

According to Reference [27], the quaternion vector can be updated as follows:

(2) $$q_{k+1}=\left[\mathrm{diag}(I,0)\cos\frac{\Delta \theta_{k}}{2}+\Delta \Theta \frac{\sin\frac{\Delta \theta_{k}}{2}}{\Delta \theta_{k}}\right]q_{k}$$

where diag(I,0) denotes a 3∗3 identity matrix. $q_{k+1}={\left[q_{1},q_{2},q_{3},q_{4}\right]}_{k+1}$ represents the quaternion vector at the (k+1)th time. $\Delta \theta_{k}=\sqrt{{\left(\Delta \theta_{k}^{x}\right)}^{2}+{\left(\Delta \theta_{k}^{y}\right)}^{2}+{\left(\Delta \theta_{k}^{z}\right)}^{2}}$. ΔΘ is calculated as follows:

(3) $$\Delta \Theta =\left[\begin{array}{cccc} 0 & \Delta \theta_{k}^{z} & -\Delta \theta_{k}^{y} & \Delta \theta_{k}^{x}\\ -\Delta \theta_{k}^{z} & 0 & \Delta \theta_{k}^{x} & \Delta \theta_{k}^{y}\\ \Delta \theta_{k}^{y} & -\Delta \theta_{k}^{x} & 0 & \Delta \theta_{k}^{z}\\ -\Delta \theta_{k}^{x} & -\Delta \theta_{k}^{y} & -\Delta \theta_{k}^{z} & 0 \end{array}\right]$$

Once the quaternions are updated, the direction cosine matrix is calculated as follows:

(4) $$C_{b,k+1}^{n}={\left[\begin{array}{ccc} q_{1}^{2}-q_{2}^{2}-q_{3}^{2}+q_{4}^{2} & 2q_{1}q_{2}-2q_{3}q_{4} & 2q_{1}q_{3}+2q_{2}q_{4}\\ 2q_{1}q_{2}+2q_{3}q_{4} & -q_{1}^{2}+q_{2}^{2}-q_{3}^{2}+q_{4}^{2} & 2q_{2}q_{3}-2q_{1}q_{4}\\ 2q_{1}q_{3}-2q_{2}q_{4} & 2q_{2}q_{3}+2q_{1}q_{4} & -q_{1}^{2}-q_{2}^{2}+q_{3}^{2}+q_{4}^{2} \end{array}\right]}_{k+1}$$

where $C_{b,k+1}^{n}$ represents the direction cosine matrix at the (k+1)th time. The function of $C_{b,k+1}^{n}$ is to convert the acceleration and angular velocity in the carrier coordinate system to the navigation coordinate system.

After the direction cosine matrices have been updated, the attitude angle can be calculated as follows [26]:

(5) $$\begin{array}{cc} \vartheta_{k+1} & =\tan^{-1}\frac{-C_{b,k+1}^{n}\left(3,1\right)}{\sqrt{{\left(C_{b,k+1}^{n}\left(3,2\right)\right)}^{2}+{\left(C_{b,k+1}^{n}\left(3,3\right)\right)}^{2}}}\\ \phi_{k+1} & =\tan^{-1}\frac{C_{b,k+1}^{n}\left(3,2\right)}{C_{b,k+1}^{n}\left(3,3\right)}\\ \psi_{k+1} & =\tan^{-1}\frac{C_{b,k+1}^{n}\left(2,1\right)}{C_{b,k+1}^{n}\left(1,1\right)} \end{array}$$

where $\vartheta_{k+1},\phi_{k+1},\psi_{k+1}$ denote the pitch, roll, and yaw at the (k+1)th time, respectively.

#### 4.1.2. Step Length

**Figure 2:**
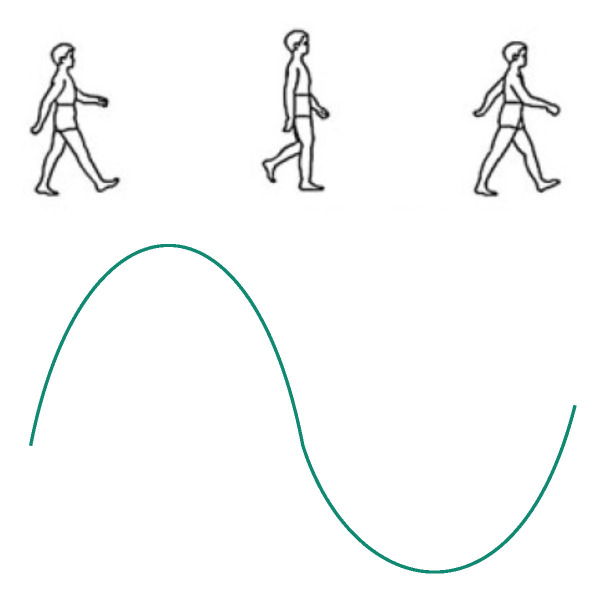
Pedestrian walking posture.

Gait-based step length estimation has been successfully applied in low-end inertial sensors [28]. Vertical displacement is periodic while a pedestrian walks, as illustrated in Figure 2.

The vertical displacement of pedestrians during walking is calculated as follows [29]:

(6) $$h\left(k\right)=-\int_{t1}^{t2}V_{z}\left(t\right)dt$$

where h(k) and $V_{z}$ denote the vertical displacement and vertical speed at the kth time, respectively.

According to the literature [29], step length can be calculated as follows:

(7) $$SL\left(k\right)=2\sqrt{-2L\int_{t1}^{t2}V_{z}\left(t\right)dt-{\left(\int_{t1}^{t2}V_{z}\left(t\right)dt\right)}^{2}}$$

where SL(k) represents the leg length at the kth time.

#### 4.1.3. The Extended Kalman Filter for Accelerometer and Gyroscope Drift

In the process of walking, when a pedestrian’s foot hits the ground, their vertical speed should be zero. Using this observation information, EKF can be constructed to filter out walking noise. The corresponding system model is written as follows [29]:

(8) $$X\left(k\right)=\left[Z\left(k\right),V_{z}\left(k\right),\mathrm{Roll}\left(k\right),\mathrm{Pitch}\left(k\right),\mathrm{Yaw}\left(k\right)\right]$$

where Z(k) and $V_{z}\left(k\right)$ represent the vertical displacement and vertical velocity, respectively; and Roll(k),Pitch(k),Yaw(k) represents the attitude angle.

The vertical velocity and vertical displacement of the transition model is as follows [29]:

(9) $$\left[\begin{array}{c} Z(k+1)\\ V_{z}\left(k+1\right) \end{array}\right]=\left[\begin{array}{cc} 1 & T_{s}\\ 0 & 1 \end{array}\right]⊗\left[\begin{array}{c} Z(k)\\ V_{z}\left(k\right) \end{array}\right]+\left[\begin{array}{ccc} 0 & 0 & \frac{T_{s}^{2}}{2}\\ 0 & 0 & T_{s} \end{array}\right]⊗\left[\begin{array}{c} a_{x}\\ a_{y}\\ a_{z} \end{array}\right]$$

where $T_{s}$ represents the sampling interval and $a_{x},a_{y},a_{z}$ represents the tri-axis acceleration, respectively.

EKF is triggered while the time moves to the moment where Zero Velocity Update (ZUPT) detects zero vertical velocity; the vertical velocity is then updated to zero. The algorithm flowchart for the EKF module is shown in Algorithm 1.
**Algorithm 1** The EKF module.**Input:** Accelerometers and gyroscopes collect acceleration and angular velocity data
Extract the vertical acceleration from acceleration.Estimate the zero-velocity point using vertical acceleration.Compensate for acceleration and angular velocity errors.Use acceleration to calculate the quaternion.Obtain the attitude angle from the quaternion.Filter noise using EKF.


#### 4.1.4. DR

Based on the step length, direction, and position at the kth time, the pedestrian position at the (k+1)th time is calculated as follows:

(10) $$\left[\begin{array}{c} x(k+1)\\ y(k+1) \end{array}\right]=\left[\begin{array}{c} x(k)\\ y(k) \end{array}\right]+SL\left(k\right)\times \left[\begin{array}{c} \sin(\psi (k))\\ \cos(\psi (k)) \end{array}\right]$$

where x(k) and y(k) denote the pedestrian’s position at the kth moment.

### 4.2. Multi-Dimensional Euclidean Distance for WiFi Matching

**Figure 3:**
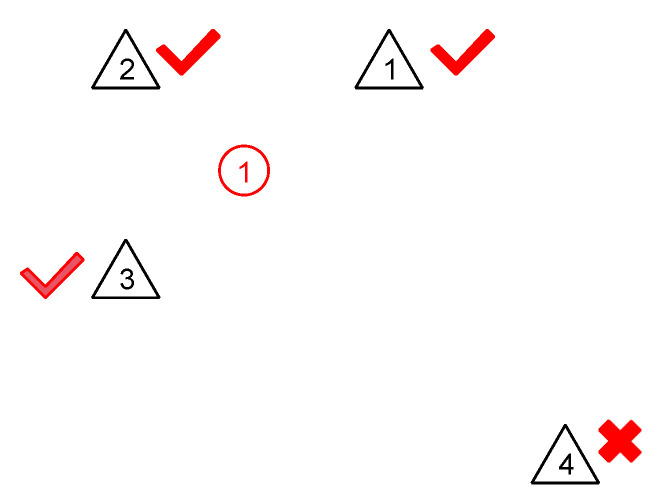
A “jump point” for WiFi fingerprint matching.

Single-point fingerprint matching is a common algorithm in WiFi fingerprint matching [10].Yet single-point matching suffers from a “jump point” problem, mainly due to large WiFi fluctuations. As shown in Figure 3, a “jump point” occurs when a pedestrian’s position is estimated. Each triangle represents the fingerprint matching position after WiFi fingerprint matching. The fourth triangle’s far distance from the other three triangles indicates an unreliable WiFi matching position.

**Figure 4:**
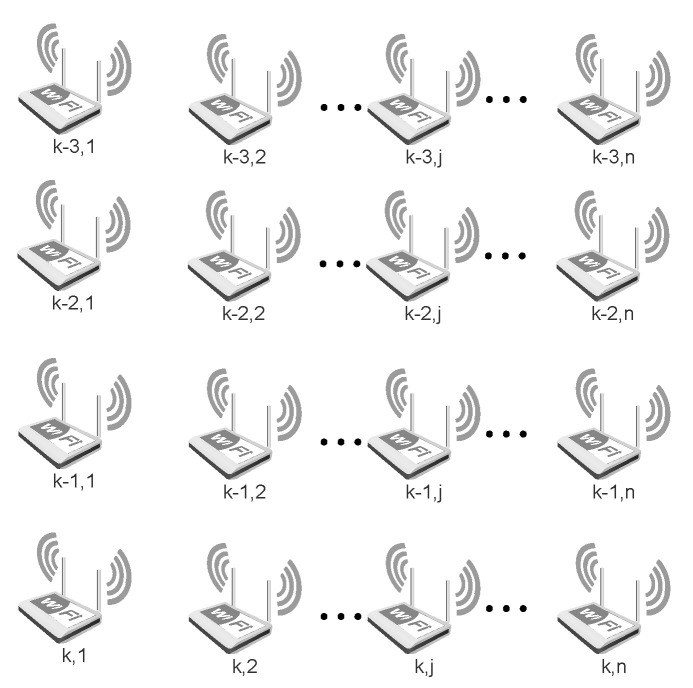
The multi-dimensional WiFi fingerprint matching.

A multidimensional fingerprint algorithm is proposed here to solve the jump point problem. By increasing the amount of historical fingerprint information and reducing the impact of single fingerprint matching, the fingerprint matching algorithm becomes more stable. In this case, *n* represents the number of WiFi access points collected simultaneously; the square represents the RSS of the access point; and *k* denotes the kth time at which the WiFi fingerprint was collected; see Figure 4.

The received signal strength (RSS) of each WiFi access point is used as an element of a one-dimensional fingerprint. Multiple access points form a one-dimensional fingerprint at the same time. Multiple access points at different times constitute multi-dimensional WiFi fingerprints. Compared with a one-dimensional WiFi fingerprint, a multi-dimensional fingerprint can effectively reduce the occurrence of “jump points" in fingerprint matching results. The traditional Euclidean distance is only suitable for one-dimensional WiFi fingerprints. For multi-dimensional WiFi fingerprints, the traditional Euclidean distance needs to be modified. Considering the multi-dimensional attribute of WiFi fingerprint, the corresponding MED is proposed, which is calculated as follows:

(11) $$d=\sum_{i=k-3}^{k}\left[\sum_{j=1}^{n}\left(RSS\left(i,j\right)-RSS_{db}\left(i,j\right)\right)\right]$$

where RSS(i,j) denotes the jth access point value at the ith time in the online stage, and $RSS_{db}\left(i,j\right)$ denotes the jth access point value at the ith time in the offline stage. After calculating the fingerprint distance, WKNN is used to estimate the WiFi fingerprint matching position. This process is described in detail in Reference [20].

### 4.3. The Adaptive Particle Filter for Pedestrian Localization

#### 4.3.1. System Equation for the Adaptive Particle Filter

Different from the Kalman filter, the particle filter provides a nonlinear system state estimation technique [30]. Because particle filter technology is independent of the system model, this filter has been widely applied in various nonlinear and non-Gaussian system models. The state equation and observation equation of the particle filter are written as follows:

(12) $$X_{k}=f\left(X_{k-1}\right)+\omega$$

(13) $$Z_{k}=h\left(X_{k}\right)+\nu$$

where f(·) and h(·) represent known processes and observation equations, respectively; $X_{k}$ and $Z_{k}$ represent the process state and the observed state at the kth moment, respectively; and ω and ν represent the process noise and observation noise, respectively.

In DR, the step length, direction, and estimated position each contain a certain error. It is assumed that the step length noise, direction change noise, and pedestrian position noise obey the Gaussian distribution of zero mean. Based on DR, the process equation for the particle filter is defined as follows:

(14) $$\begin{array}{cc} X_{k} & =f(X_{k-1})+\omega \\ \; & =\left[\begin{array}{c} L_{k-1}\\ \theta_{k-1}\\ x_{k-1}\\ y_{k-1} \end{array}\right]+\left[\begin{array}{c} \Delta L\\ \Delta \theta \\ L_{k}\cos\left(\theta_{k}\right)\\ L_{k}\sin\left(\theta_{k}\right) \end{array}\right]+\omega \end{array}$$

where $L_{k-1}$ and $\theta_{k-1}$ represent the step length and direction at the (k−1)th time. $\left(x_{k-1},y_{k-1}\right)$ represents the pedestrian’s estimated position at (k−1)th time; and ΔL and Δθ represent the pedestrian’s step length and direction change, respectively.

The WiFi positioning system can provide positioning results as a pedestrian walks. Combined with DR, the observation equation is written as follows:

(15) $$Z_{k}=h\left(X_{k}\right)+\nu =\left[\begin{array}{c} x_{DR}\\ y_{DR} \end{array}\right]-\left[\begin{array}{c} x_{WIFI}\\ y_{WIFI} \end{array}\right]+\nu$$

where ${\left[x_{DR},y_{DR}\right]}^{T}$ represents the positioning result of DR and ${\left[x_{WIFI},y_{WIFI}\right]}^{T}$ represents the positioning result of WiFi fingerprint matching.

#### 4.3.2. Particle Weight Technique

In nonlinear systems, it is difficult to discern an analytical solution for a posteriori particle filter distribution. One feasible approach is to use the approximate solution instead of the analytical solution. A large number of particles are used to describe a posteriori distribution. A resampling technique has been introduced [31] to mitigate the sampling problem in a posteriori distribution:

(16) $$q\left(X_{0:k}|Z_{1:k}\right)=q\left(X_{0:k-1}|Z_{1:k-1}\right)q\left(X_{k}|X_{0:k-1},Z_{1:k}\right)$$

The recursive formula for calculating the importance weight of each generation of particles is:

(17) $$\begin{array}{cc} {\omega 1}_{k}^{i} & =\frac{p\left(X_{0:k}|Z_{1:k}\right)}{q\left(X_{0:k}|Z_{1:k}\right)}\\ \; & \propto {\omega 1}_{k-1}^{i}\frac{p\left(Z_{k}|X_{k}\right)p\left(X_{k}|X_{k-1}\right)}{q\left(X_{k}|X_{0:k-1},Z_{1:k}\right)}\\ \; & ∼\frac{1}{{\left(2\pi \right)}^{m/2}{\left|R\right|}^{1/2}}\exp\left(\frac{-{\left[Z^{*}-h\left(X_{k,i}^{-}\right)\right]}^{T}R^{-1}\left[Z^{*}-h\left(X_{k,i}^{-}\right)\right]}{2}\right) \end{array}$$

where $Z^{*}$ represents the estimated observation; $X_{k,i}^{-}$ represents the priori estimated state vector; *m* represents the vector dimension; and *R* represents the observation noise.

**Figure 5:**
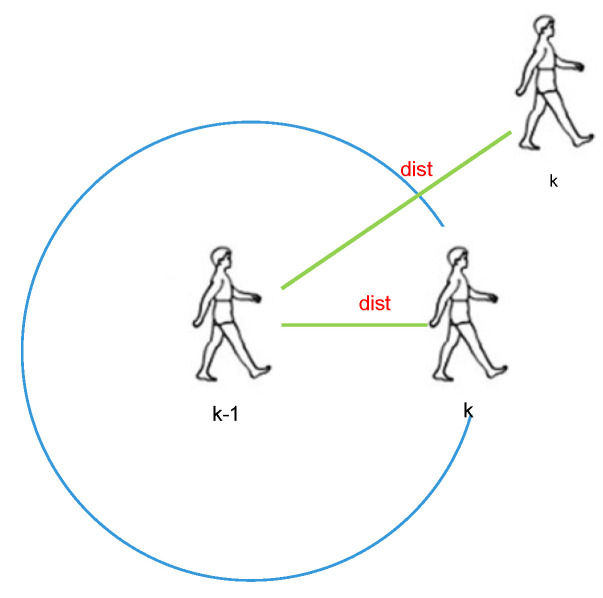
Schematic diagram of pedestrian walking relationship.

In the improved particle filter, the weight of particles depends not only on the observation but also on the distance between the particles at the current moment and the historical moment. Considering that the pedestrian walking speed is usually 1 m/s to 1.5 m/s, the pedestrian position is constrained by the walking speed. The position of the previous moment is the center of the circle, and the distance between the position of the current moment and the position of the previous moment is the radius of the circle, forming a circle, as shown in Figure 5. As you can see from the figure, when the particles are in the circle, the weight of the particles should be higher. When the particles are outside the circle, the farther away the particles are, the lower the weight of the particles is. As such, an adaptive factor is proposed to calculate particles’ weight:

(18) $$\begin{array}{ccc} dist^{i} & = & ∥{\left[\begin{array}{c} x_{est}\\ y_{est} \end{array}\right]}_{k}^{i}-\left[\begin{array}{c} \hat{x}\\ \hat{y} \end{array}\right]∥ \end{array}$$

(19) $$\begin{array}{ccc} {\omega 2}_{k}^{i} & = & \frac{1}{1+e^{-dist^{i}}} \end{array}$$

where ${\left[\begin{array}{c} x_{est}\\ y_{est} \end{array}\right]}_{k}^{i}$ represents the ith particle position at the kth moment. $\left[\begin{array}{c} \hat{x}\\ \hat{y} \end{array}\right]$ represents the previous moment’s estimated particle position. ∥·∥ represents the norm. dist represents the distance between the position of the previous moment and the position of the current moment. ω2 represents the particle weight.

Upon combining the two particle weight calculation methods, the new particle weight ω can be calculated as follows:

(20) ω=ω1∗ω2

#### 4.3.3. Map Assistance

In the offline phase, floor structure map information is stored in the map database in advance. Newly generated particles need to be tested for effectiveness in the online phase. If a particle crosses the wall, then its weight is set to zero; if a particle does not cross the wall, then its original weight is used. The map assistance algorithm is described further in [22].

## 5. Experimental Results

### 5.1. Experimental Scene

**Figure 6:**
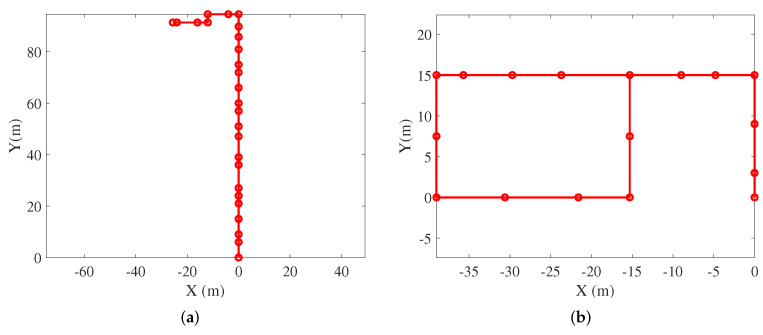
The real trajectories in the office building A and B. (**a**) The office building A; (**b**) The office building B.

The walking experiment was carried out in office buildings A and B. The test scene included metal, wood, and concrete structures. An adult male with a height of 1.7 m holds a smartphone to collect data and walks at a speed of 1 m/s to 1.4 m/s. The tester opens the software at the starting point and clicks the “Start Saving a Log File” button to start collecting data. Click the “Stop Saving” button at the endpoint to save the data. The mapper’s actual position was measured using a laser rangefinder. In the offline phase, the tester walked from the starting point to the end point while holding the smartphone while the smartphone’s WiFi chip collected the RSS value of AP. The WiFi fingerprint database was established based on the laser rangefinder and the corner of the used corridor. In the online phase, each test pedestrian held a smartphone which obtained the RSS value of AP at normal walking speed. The experimental walking distance in office buildings A and B was 123 m and 138 m, respectively. The real trajectory is shown in Figure 6. The circle represents the reference point. These reference points are used to calculate errors and evaluate the performance of the algorithm.

### 5.2. Acceleration and Gyroscope Data

**Figure 7:**
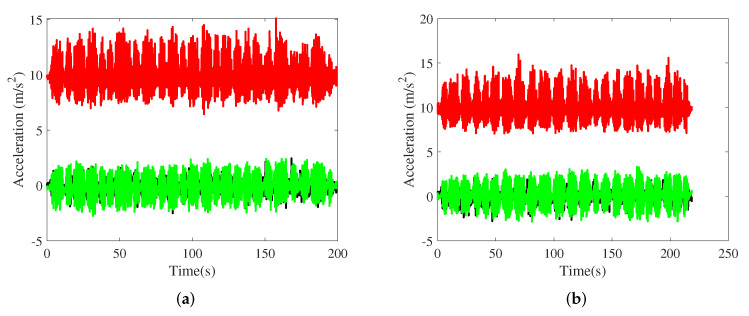
Acceleration in the office building A and B. (**a**) The office building A; (**b**) The office building B.

**Figure 8:**
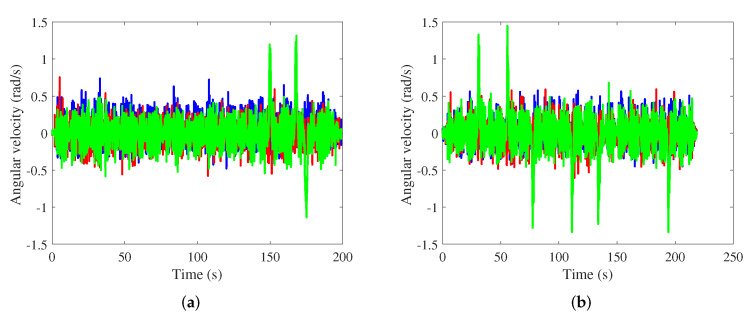
Angular velocity in the office building A and B. (**a**) Office building A; (**b**) Office building B.

The parameters of the smartphone are shown in Table 1, and the sampling frequency is set to 50Hz. Acceleration and angular velocity data obtained in office buildings A and B are depicted in Figure 7 and Figure 8, respectively. The acceleration was approximately periodic. The gait model was adopted to obtain pedestrians’ step length. Angular velocity clearly increased at the turning point.

### 5.3. WiFi Fingerprint Database

Limited by the size of the smartphone, the battery capacity is limited. To reduce battery consumption, smartphones scan WiFi relatively less frequently. Taking a Nexus 5X used to construct a fingerprint database as an example, the sampling frequency is 0.25 Hz. Spatial interpolation is an effective way to improve the spatial resolution of the WiFi fingerprint database. The spatial resolution of the fingerprint is improved to 0.1Hz by interpolation. The positioning errors of the original fingerprint database and the interpolated fingerprint database are shown in Table 2.

### 5.4. Multidimensional WiFi Fingerprint

Different values of WiFi fingerprint dimensions were simulated; the average error is listed in Table 3. The minimum position error was obtained via MED when using two-dimensional WiFi fingerprints. The error caused by abnormal fingerprints could be reduced by expanding the fingerprint dimensions.

### 5.5. Comparison between Multi-Dimensional Euclidean Distance and Euclidean Distance

**Figure 9:**
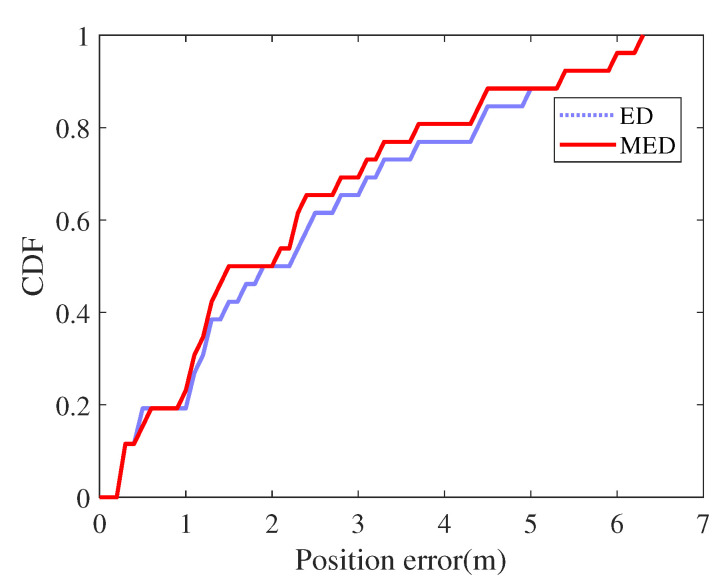
CDF of errors.

Figure 9 shows the relationship between position error and cumulative distribution function (CDF). Compared with traditional ED, MED uses multidimensional fingerprints to effectively reduce the error caused by abnormal fingerprints.

### 5.6. Performance Analysis of an Adaptive Particle Filter

**Figure 10:**
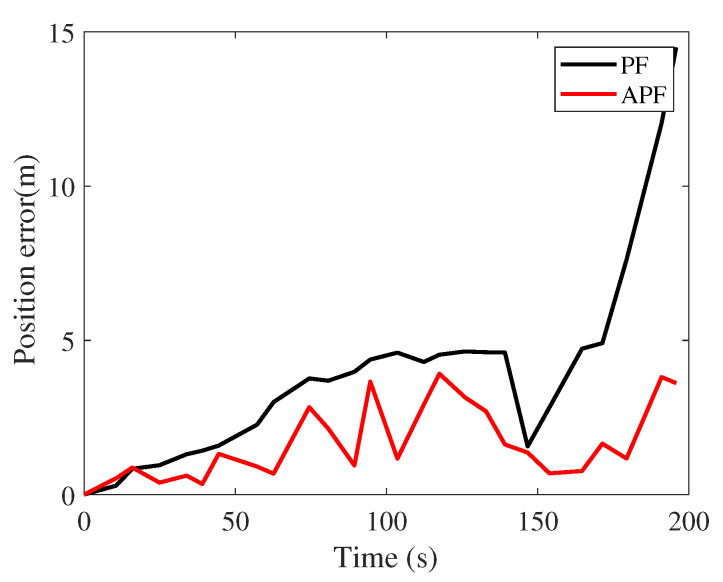
Position errors.

Figure 10 displays the position error of the traditional particle filter (PF) and adaptive PF. The APF positioning error tended to be lower than that of the traditional PF. APF uses map topology to constrain the particle range. In addition to being determined observationally, the particle weight is related to the distance between particles’ current and historical positions: the farther the distance, the lower the reliability.

### 5.7. Office Building A

**Figure 11:**
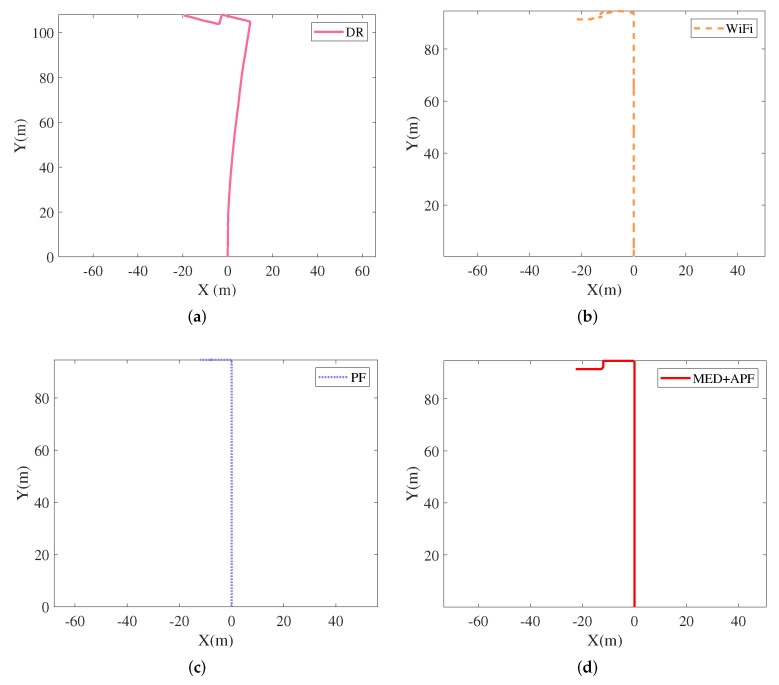
Positioning trajectories with different strategies. (**a**) DR; (**b**) WiFi; (**c**) PF; (**d**) MED+APF.

**Figure 12:**
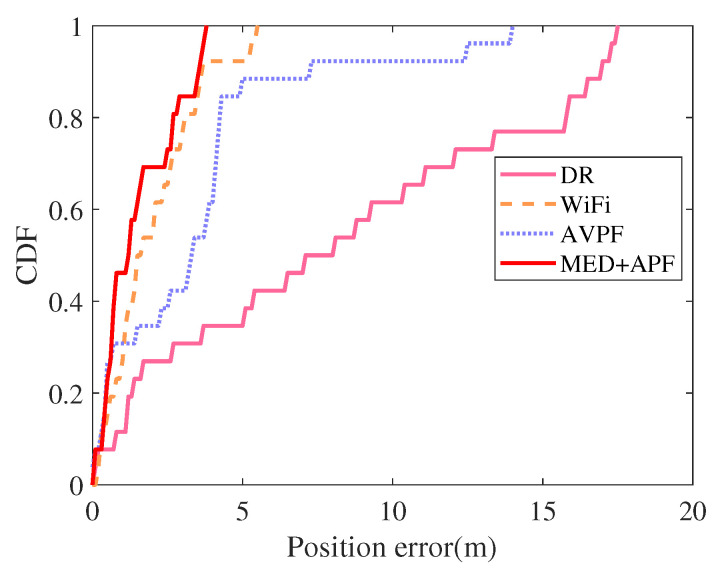
CDF of errors.

Pedestrians walked on the predetermined trajectory in office building A with a Nexus 5 smartphone in hand. Figure 11 and Figure 12 show the positioning trajectories and cumulative distribution function (CDF) for DR, WiFi, PF, and MED+APF, respectively. As listed in Table 4, the average error for DR, WiFi, PF, and MED+APF was 8.03 m, 1.98 m, 3.45 m, and 1.51 m, respectively; the respective root mean square error was 10.02 m, 2.43 m, 4.82 m, and 1.92 m. The positioning trajectory was initially along the y-axis with a high positioning accuracy. As the pedestrians’ walking time lengthened, the positioning trajectory shifted well to the right, and the position error increased. DR reflected cumulative error in that the longer the pedestrian’s walking time, the larger the error. No cumulative error was found in WiFi fingerprint matching; however, fingerprint mismatching occurred. PF was thus adopted to integrate DR and WiFi. Aiming at the fingerprint mismatching caused by a “jump point” fingerprint, the multi-dimensional Euclidean distance algorithm introduces historical fingerprint information to reduce the position error. To solve the problem of particle degradation, the historical position information is introduced, and APF uses the adaptive factor to improve the weight of high trust particles and reduce the weight of low trust particles. These algorithms collectively improve the accuracy of pedestrian localization.

### 5.8. Office Building B

**Figure 13:**
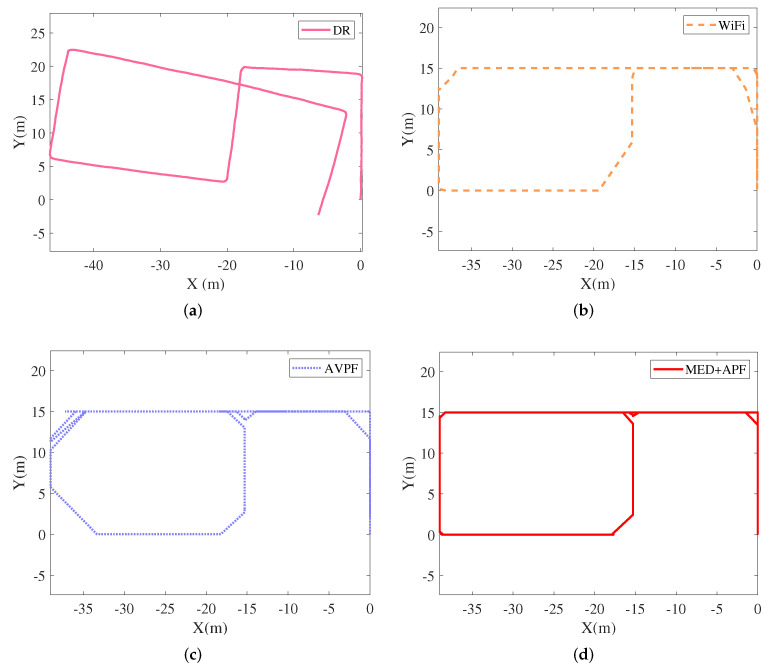
Positioning trajectories with different strategies. (**a**) DR; (**b**) WiFi; (**c**) AVPF [32]; (**d**) MED+APF.

**Figure 14:**
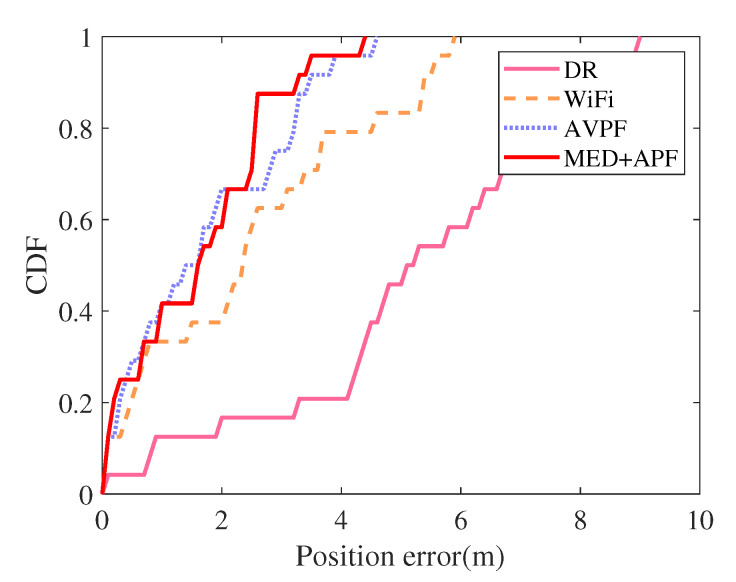
CDF of errors.

An adult man held a Nexus 5X smartphone that collected acceleration, angular velocity, and WiFi signals. Auxiliary value particle filter (AVPF) uses maps to determine particle weights [32]. If the particle crosses the wall, set its corresponding particle weight to zero. DR, WiFi, AVPF [32], and MED+APF positioning trajectories appear in Figure 13. the CDF of error is listed in Figure 14. DR exhibited an obvious cumulative error: the longer the running time, the greater the position error. The longer the running time, the greater the position error. The pedestrians’ position error was small when first walking, yet as their walking time increased, the position error deviated clearly from their actual position. The WiFi signal also fluctuated because fingerprint acquisition and matching occurred at different times, leading to mismatched WiFi fingerprint matching. Mismatching caused a particularly prominent error in the corridor corner. AVPF uses map assistance to detect particles’ validity and sets the particle weight to zero if the particle crosses the wall. Meanwhile, the map-matching algorithm further reduces position error. By adding dimension-related information from historical WiFi fingerprints, this work first employed MED to calculate the distance between WiFi online fingerprints and fingerprints in the fingerprint database. Then, considering the distance relationship between particles’ current and historical positions, an APF algorithm was proposed by introducing an adaptive factor.

The DR, WiFi, AVPF [32], and MED+APF position error appear in Table 5. Compared with DR, WiFi, and AVPF [32], the average error of MED+APF declined by 65.23%, 27.05%, and 13.17%, respectively; the root mean square error fell by 62.01%, 29.97%, and 26.37%, respectively. The best positioning algorithm was that proposed in this paper: MED+APF.

## 6. The Significance of the Proposed Method

This paper proposes a positioning algorithm that combines inertial sensors and WiFi based on existing WiFi access points. It is simple to update the software of the smart terminal, which is popular among consumers. Simultaneously, the multi-dimensional WiFi fingerprint algorithm is advantageous in reducing the occurrence of fingerprint mismatching and improving the accuracy of the fingerprint matching algorithm. Finally, the addition of DR historical position information alleviates the particle degradation problem.

## 7. Conclusions

The INS is less influenced by external interference and possesses high short-term positioning accuracy. However, DR error has a cumulative effect and larger long-term position error. Fingerprint mismatching occurred despite the lack of cumulative error, associated with WiFi fingerprint matching. This paper proposes an MED and an APF. MED uses multidimensional WiFi fingerprints to reduce fingerprint mismatching; APF uses an adaptive factor to increase the weight of highly trusted particles while reducing the weight of less trusted particles. These two methods can effectively reduce indoor position error.

Certain problems remain to be addressed in future work. First, the proposed algorithm must be applied in additional scenario tests to determine its robustness. Second, the particle filter requires extensive calculation; it is therefore necessary to consider how to reduce this computational burden. Third, more sensors can be integrated in the future to further reduce position error.

## Figures and Tables

**Table 1:** The Parameters of Sensor.

Sensor	Model	Resolution	MaxRange
Acceleration	BMl160	0.0023942017 m/s${}^{2}$	78.4532 m/s${}^{2}$
Gyroscope	BMl160	0.0010652645 rad/s	34.906586 rad/s

**Table 2:** Position errors (m).

	Average Error
The original fingerprint database	5.77
The interpolated fingerprint database	1.98

**Table 3:** Position errors (m).

WiFi Fingerprint Dimension	1	2	3	4
Average error	2.43	2.28	2.29	2.41

**Table 4:** Position errors of DR, WiFi, PF, and MED+APF (m).

Position Error	Average Error	Root Mean Square Error
DR	8.03	10.02
WiFi	1.98	2.43
AVPF	3.45	4.82
MDE+APF	1.51	1.92

**Table 5:** Position errors of DR, WiFi, AVPF [32], and MED+APF (m).

Position Error	Average Error	Root Mean Square Error
DR	5.12	5.66
WiFi	2.44	3.07
AVPF	2.05	2.92
MED+APF	1.78	2.15

## Data Availability

GitHub repository. Available online at: https://github.com/Localization-IMU.

## Abbreviations

The following abbreviations are used in this manuscript:

INS	Inertial navigation system
HDR	Heuristic drift reduction
HDE	Heuristic drift elimination
LD	Linear dichroism
PF	Particle filter
DR	Dead reckoning
APF	Adaptive particle filter
MED	Multi-dimensional Euclidean distance
WKNN	Weighted K nearest neighbor
CDF	Cumulative Distribution Function
